# Effects of Cold Plasma on Food Quality: A Review

**DOI:** 10.3390/foods7010004

**Published:** 2018-01-01

**Authors:** Shashi K. Pankaj, Zifan Wan, Kevin M. Keener

**Affiliations:** Center for Crops Utilization Research, Iowa State University, Ames, IA 50011, USA; shashiskp143@gmail.com (S.K.P.); zwan@iastate.edu (Z.W.)

**Keywords:** cold plasma, food quality, physical quality, chemical quality

## Abstract

Cold plasma (CP) technology has proven very effective as an alternative tool for food decontamination and shelf-life extension. The impact of CP on food quality is very crucial for its acceptance as an alternative food processing technology. Due to the non-thermal nature, CP treatments have shown no or minimal impacts on the physical, chemical, nutritional and sensory attributes of various products. This review also discusses the negative impacts and limitations posed by CP technology for food products. The limited studies on interactions of CP species with food components at the molecular level offers future research opportunities. It also highlights the need for optimization studies to mitigate the negative impacts on visual, chemical, nutritional and functional properties of food products. The design versatility, non-thermal, economical and environmentally friendly nature of CP offers unique advantages over traditional processing technologies. However, CP processing is still in its nascent form and needs further research to reach its potential.

## 1. Introduction

Over the past decade, cold plasma (CP) has gained significant interest for use as a non-thermal technology for food processing. The novelty of this technology lies with its non-thermal, economical, versatile and environmentally friendly nature. The applications of CP for food industries have been demonstrated for food decontamination [1], enzyme inactivation [2], toxin removal [3], food packaging modifications [4] and waste water treatment [5]. Particularly for food processing, CP has been shown to be effective against major food-borne pathogenic microorganisms such as *Escherichia coli* [6], *Salmonella typhimurium* [7], *Staphylococcus aureus* [8], and *Listeria monocytogenes* [9].

Quality, both in terms of objective product characteristics and subjective consumer perception, is an essential factor for the success of any food product [10]. Thermal processing of food has been in use for more than two centuries and is still the major food processing technique used in the food industries [11]. The use of severe heat leads to undesirable effects such as change in color, texture, loss of nutrients etc., motivating researchers to explore non-thermal alternatives for food processing. CP is one of the non-thermal technologies which has shown significant potential in this regard. CP’s inactivation of pathogenic and spoilage microorganisms could result in minimally processed, safe food products with extended shelf-life. However, most of the published research has been focused on microbial decontamination, with limited studies on the impact of CP processing on quality attributes.

The aim of this review is to provide a brief description of the CP technology and plasma processing for food industries and analyze the impact of CP processing on the quality attributes of various food products.

## 2. Plasma Physics and Sources

Plasma is a quasi-neutral ionized gas state composed of ions, free electrons, atoms and molecules in their fundamental or excited states with a net neutral charge [12]. Based on the thermal equilibrium, plasma can be classified as thermal and low-temperature plasma. In thermal plasma, all species exist in a thermodynamic equilibrium (e.g., arc plasma; electron temperature ≈ heavier species temperature ≈ 10,000 K) whereas, in the other class, the temperatures of all species are the same in localized areas in the plasma. Low temperature plasma can be further subdivided into thermal plasma (quasi-equilibrium plasma), which are in a local thermal equilibrium state, and non-thermal plasma (non-equilibrium plasma), where species are in thermal non-equilibrium (e.g., glow discharges; electron temperature ≈ 10,000–100,000 K, heavier species temperature ≈ 300–1000 K) [2]. The non-thermal plasma, where electrons and heavier species are in thermal non-equilibrium, is referred to as cold plasma in this review. Depending on the pressure conditions, plasma can also be classified as high-pressure, atmospheric pressure and low-pressure plasma. In atmospheric pressure plasma, plasma is generated at the normal atmospheric pressure, eliminating the need for cost-intensive reaction chambers to maintain pressure.

**Figure 1:**
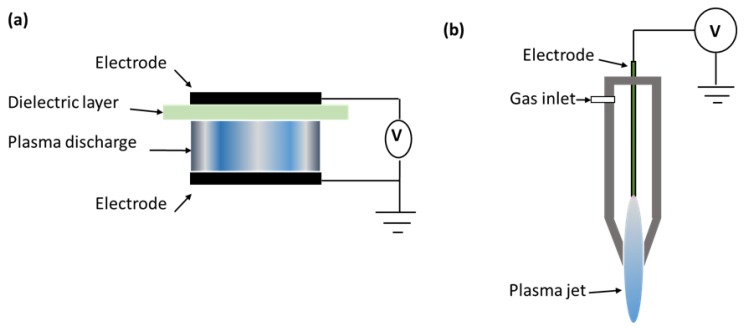
Schematic diagram of (**a**) dielectric barrier discharge; (**b**) plasma jet system. Adapted from [13].

Plasma can be generated using any kind of energy which can ionize the gases, such as electrical, thermal, optical (UV light), radioactive (gamma radiation) and X-ray electromagnetic radiation. However, electric or electromagnetic fields are widely used for CP generation [13]. The versatility of CP generation sources offers unique designs which are compatible with current food industry equipment. With regards to food processing, dielectric barrier discharge and jet plasma are most commonly used (Figure 1). Dielectric barrier discharge (DBD) devices consist of two metal electrodes, where at least one of these electrodes is covered with a dielectric barrier. Dielectric barriers act as a stabilizing material, avoiding any arc transition, and help in creating a large number of micro-discharges for homogeneous treatments. Plasma jet devices consist of two concentric electrodes, where the inner electrode is typically connected to a radio frequency power at high frequency causing ionization of the working gas, which exits the nozzle and gives a ‘jet-like’ appearance. Further details of these systems are available elsewhere [14,15,16].

However, at this point, it is worth mentioning the ‘in-package’ plasma mode of processing, which has demonstrated huge potential for the food processing industry [2]. In this mode, packaged food is placed between the electrodes to ionize the headspace gas to generate reactive species. The advantages of this mode of processing are easy scalability into a continuous system, enhanced antimicrobial efficacy and prevention of cross-contamination.

From a food processing perspective, plasma source, electrode design, pressure, voltage, treatment time, distance between electrodes and reactive gas all play important roles in determining the gas speciation, reactive species concentration, discharge characteristics and overall efficiency of the process. It is important to mention that the differences in various systems also present a huge challenge for researchers to compare and interpret the published results across different experimental setups. 

## 3. Physical Quality

CP is generally considered as a tool for surface treatments. In fact, CP has been used by the polymer and packaging industries for decades for surface modification and functionalization of polymers [17]. However, during processing of food, food products might be placed in strong electric field and are subjected to numerous reactive gas species that could affect physical quality attributes such as color and texture, which will be discussed in the next section. A summary of effects of CP on food products has been presented in Table 1.

### 3.1. Color

Color of food products is an important attribute which has a direct effect on consumer perception and hence the success of any product. The color of food products are mostly due to presence of pigments (natural or synthetic) and chemical reactions (enzymatic or non-enzymatic). Any undesirable change in the color of food products due to processing technique will be a big obstacle for its acceptability.

Varying effects of CP treatments on the color of fresh fruits and vegetables have been reported depending on the severity of treatment conditions. Various researchers reported no significant loss of color after CP treatments of strawberry, apples, kiwifruit, cherry tomatoes, lettuce and carrots [6,26,29,30,33]. Some researchers reported minor changes after the CP treatments [27,34]. In some cases, such as blueberry, Sarangapani, O’Toole, Cullen and Bourke [24] and Lacombe, Niemira, Gurtler, Fan, Sites, Boyd and Chen [25] reported loss of color at higher treatment times. Similarly, total color difference after CP treatment of fruit juices were also found minimal and not perceptible by naked eyes [18,22]. Amini et al. [57] also observed loss in quality for saffron after increasing input voltage and addition of oxygen in the working gas. The changes in the color could be due to the partial degradation of pigments such as chlorophyll and anthocyanin, as reported in some studies [25,29]. Overall, these results demonstrate that CP processing has a minimal effect on the color of food products at lower treatment times. The product type (whole or cut, solid or liquid), plasma treatment parameters (input voltage, time, power, working gas) and storage conditions are some of the critical factors affecting the color.

CP processing was also reported to lead to certain desirable effects on the color of a few food products. Thirumdas, Saragapani, Ajinkya, Deshmukh and Annapure [41] have reported an increase in the brightness and whiteness index of brown rice after plasma treatment. In another study, Yong et al. [58] have used CP in the manufacturing of pork jerky without adding sodium nitrite. They used specific plasma processing parameters to achieve similar redness/color in the pork jerky without using any chemical nitrite additive. These studies extends the current area of research for development of new products with CP technology, which will be natural and free from chemical additives.

### 3.2. Texture

Many of the reported studies suggest the retention of texture of food products after CP processing. In case of fresh fruits and vegetables, no significant difference was observed after CP treatment of strawberry, apple, melons and cherry tomatoes [26,30,32,33]. However, a decrease in firmness was reported after CP treatment of blueberries [24,25]. The softening of the blueberries was attributed to the mechanical damage due to the high air-flow rates of the plasma jet and the temperature rise during the treatment. In another study on CP treatments of strawberry in modified atmosphere packaging, the firmness retention was found to be better in a high-oxygen environment (65% O_2_ + 16% N_2_ + 19% CO_2_) than a nitrogen-rich environment (90% N_2_ + 10% O_2_) [27]. This study demonstrates that plasma gas is an important factor influencing the firmness of treated products. Similar increased texture retention under high oxygen atmosphere and ozone treatments have also been reported in the literature [59,60]. They suggested that the enhanced firmness retention is due to the reduction in ripening rate as a stress response to high oxygen atmosphere.

CP treatment of grains and legumes resulted in a decrease in hardness and chewiness [40,41,42]. These groups also reported a decrease in soaking/cooking time for the plasma-treated products, which was deemed desirable for the industries. In another study on CP treatment of wheat flour, Misra, Kaur, Tiwari, Kaur, Singh and Cullen [45] reported an increase in the peak integral, elastic modulus, viscous modulus and dough strength. They also reported the effect of CP on the secondary structure of flour proteins. These studies highlights the potential of CP technology in processing of food ingredients for tailor-made visco-elastic properties.

## 4. Chemical Quality

Plasma chemistry is a complex science involving numerous species in a myriad of chemical reactions occurring in different time scales [13]. For example, air plasma involves over 75 different chemical species in almost 500 chemical reactions, making it more complex to understand their interaction with food components. However, plasma reactive species are considered to be the major factor for all the observed changes in the chemical quality attributes of the treated products, which are discussed in the following sections. It is worth noting that plasma reactive species are largely dependent on the gas used for plasma generation, making this one of the most critical factors for chemical changes.

### 4.1. pH and Acidity

pH and acidity are a closely regulated quality attribute in most of the processed food products. Any drastic change could lead to an undesirable impact on the taste, texture and shelf life of the food. However, in the case of fresh fruits and vegetables, there are significant variations due to differences in cultivation practices, varietal differences, environmental parameters etc. 

There are several reported studies where CP treatment has been shown to change the pH of food products [19,42]. The pH and acidity changes after plasma treatment were mostly attributed to the interaction of plasma reactive gases with the moisture present in the food products. In solid food products, plasma species reacts with the surface water, forming acidic compounds only on the surface while, in liquid products, effects were more pronounced. Oehmigen et al. [61] reported the formation of nitric acid induced by reactive nitrogen species such as NO as the reason for acidification in air plasma treatments. However, many researchers also reported no pH effect of CP treatments in food products with buffering capacity [18,21]. These results indicate that the effects of plasma on the pH of complex food matrices are affected by several factors such as buffering capacity, physiological activity of the living tissues, and the possibility of the liquid emanating from the damaged tissues on the surface washing off the acids on the surface [62].

### 4.2. Protein and Enzymes

The effect of CP on the protein and enzymes in food model food systems has been reviewed recently [2]. The effects of CP on various food enzymes are summarized in Table 2. The mechanisms of protein denaturation by CP could be due to the interaction of plasma reactive species with amino acids [63] and secondary structure due to loss of α-helix and β-sheet [64]. Factors like the type of protein/enzyme, type of plasma, reactive gas, processing parameters, sample volume and enzyme media play an important role on the protein denaturation and enzyme inactivation by CP. Although the enzyme inactivation could serve as an important tool for food industries, some challenges such as optimized processing parameters, better understanding of inactivation mechanisms and protective effects of different food components [65] need to be addressed.

The effects of CP in muscle protein were studied in fresh mackerel, where it resulted in a decrease in immobilized water located in the protein-dense myofibrillar network [56]. Another study on wheat flour also suggested changes in protein structure due to oxidation of sulfhydryl groups and formation of disulphide bonds, affecting its structural and functional properties.

### 4.3. Carbohydrates

Carbohydrates play an important role in defining and maintaining the quality of different food products. CP treatment of cashew apple juice resulted in the degradation of all the reducing sugars, such as fructose and glucose and non-reducing sucrose [20]. They also reported an increase in sucrose content after long exposure to CP, which they attributed to the degradation of the oligosaccharides with a high degree of polymerization. A similar decrease in the fructose, increase in the sucrose and degradation of oligosaccharides with a high degree of polymerization was also reported after CP treatment of prebiotic orange juice [19]. The studies suggest ozonolysis to be the main route of degradation causing the cleavage of glycoside bonds, leading to de-polymerization of the macromolecule and the oxidation of functional groups to form carbonyl and carboxyl compounds, lactones, hydroperoxides and CO_2_ [19,69].

The effect of CP on polysaccharides has been mainly focused on starch in legume and grain products. An increase in the water uptake rate in black gram was reported by Sarangapani, Devi, Thirumdas, Trimukhe, Deshmukh and Annapure [40], which they attributed to the surface etching and increase in water binding sites due to fragmentation of starch and protein by plasma reactive species. The same group also reported a decrease in cooking time of brown rice, indicating the incorporation of polar groups between the starch molecules [41]. They also reported an increase in degree of gelatinization after plasma treatment. In another study on rice starch, Thirumdas, Trimukhe, Deshmukh and Annapure [46] reported a decrease in the amylose content, gelatinization temperature, pasting temperature, retrogradation tendency and degree of hydrolysis. Overall, it could be concluded that CP treatment lead to de-polymerization and cross-linking of starch affecting its structural, functional and rheological properties.

### 4.4. Vitamins

The sensitivity of vitamins to different processing techniques is essential to preserve the nutritional properties of the food products. While some vitamins, such as riboflavin (B2), pyridoxine (B6) and biotin, are usually stable, others, such as Thiamin (B1) and vitamins A, C and E, are relatively labile [70]. Most of the reported studies on CP treatment of food products have only focused on vitamin C (ascorbic acid) stability.

Most of the studies on CP treatment of whole fruits and vegetables have reported no significant reduction in ascorbic acid content after plasma treatment. Ramazzina, Berardinelli, Rizzi, Tappi, Ragni, Sacchetti and Rocculi [29], Oh, Song and Min [23] and Song et al. [71] reported no significant effect on ascorbic acid in kiwifruit, radish sprout and lettuce, respectively. However, up to 4% reduction in ascorbic acid content was observed after plasma treatment of cut fruits and vegetables [34]. The reduction in ascorbic acid was also observed after CP treatment of orange juice [18] and cashew apple juice [20]. The degradation of ascorbic acid could be attributed to the reaction with ozone and other oxidizing plasma species during the processing. Sample type (whole/cut), processing time and plasma gas were critical factor for ascorbic acid degradation. However, it is important to emphasize the need for further studies to analyze the effects of CP on other vitamins in the food products along with the mechanism of degradation.

### 4.5. Lipids

Lipid oxidation is a major concern for muscle foods, which could lead to undesirable changes in the color, taste, odor and shelf life. Lipid oxidation is a complex process involving free radical chain mechanisms forming fatty acyl peroxides or other oxidation products [72]. Thiobarbituric acid reactive substance (TBARS) and peroxide value (PV) are commonly employed to measure lipid oxidation. Since CP is often considered as an advanced oxidation process, it is essential to analyze its influence on the lipids present in the muscle foods.

No significant effect on lipid oxidation were observed after CP treatment in fresh and frozen pork [49], beef jerky [8] and raw pork [54]. However, Jayasena, Kim, Yong, Park, Kim, Choe and Jo [51] reported an increase in lipid oxidation in fresh pork and beef after treating it for an extended time period of 10 min. An increase in lipid oxidation was also reported in pork loin, when it was treated with an oxygen-containing plasma gas. Recently, Albertos, Martin-Diana, Cullen, Tiwari, Ojha, Bourke, Álvarez and Rico [56] have reported that CP treatment led to a significant lipid oxidation in fresh mackerel fillets. They observed an increase in PV from 6.89 to 37.57 meq. active oxygen/kg lipids and dienes from 1.42 to 5.56 mmol of hydroperoxides/kg lipid after plasma treatment at 80 kV for 5 min. They also observed a decrease in oleic acid (C18:1, n-9) and eicosapentaenoic acid (C20:5, n-3) after plasma treatments. Recently, Sarangapani et al. [73] have shown that cold plasma oxidation of lipids follow the Criegee mechanism. They also identified typical oxidation products in model dairy and meat fat matrices as ozonides, aldehydes (hexanal, pentenal, nonanal and nonenal) and carboxylic acids (9-oxononanoic acid, octanoic acid, nonanoic acid), along with hydroperoxides (9- and 13-hydroperoxy-octadecadienoylglycerol species). The available studies on the effects of CP on lipids in different food products are very limited. However, based on the reported studies, treatment time and plasma gas could be considered as critical factors affecting lipid oxidation. 

Yepez and Keener [74] reported a novel application of CP treatment recently. They showed the potential of hydrogen plasma to be used for the manufacturing of partially hydrogenated soybean oil without any trans-fatty acid. CP technology has shown unique advantages over the current hydrogenation processes as it can be performed at room temperature, under atmospheric pressure without any catalyst. Although this approach demonstrates an alternative to the traditional catalytic hydrogenation, further research is needed to optimize the treatment process and evaluate the performance of partially hydrogenated oil made from CP.

### 4.6. Antioxidant Activity

Although antioxidant activity is not a direct quality attribute used in the food industries, it is a close indicator of various polyphenols, flavonoids and flavanols present in the food products. The antioxidant effects of phenolic compounds could be due to their redox properties, which include possible mechanisms such as free-radical scavenging activity, transition metal-chelating activity and singlet-oxygen quenching capacity [75]. The antioxidant activities in food are generally analyzed using 3-ethyl-benzothiazoline-6-sulfonic acid (ABTS) radical scavenging activity, oxygen radical absorbance capacity (ORAC), 2,2-diphenyl-1-picrylhydrazyl (DPPH) scavenging activity, and ferric reducing ability of plasma (FRAP) assay.

The reported results on the effects of CP treatment on the phenolic contents of the food products have a wide degree of variation. A decrease in the total phenols was reported in orange juice [19], white grape juice [21], and lamb’s lettuce [38]. No significant effect in apples [31] but a significant increase in cashew apple juice [20] and blueberries [24] were also reported. These differences in the reported studies highlights the research needed to better understand the effects of CP on polyphenols at a molecular level.

No significant changes in the antioxidant capacity after CP treatment were reported in radish sprouts, kiwifruits, red chicory and onion powder [23,29,36,47]. Some studies have shown a reduction in antioxidant activity after CP treatments in apples, white grape juice, and cashew apple juice on an extended exposure [20,21,31]. Almeida, Cavalcante, Cullen, Frias, Bourke, Fernandes and Rodrigues [19] reported a reduction in the antioxidant capacity of prebiotic orange juice after direct mode of plasma treatment whereas insignificant effects were reported when treated under indirect mode. These studies show that the type of food products, plasma generation source, mode of exposure and treatment parameters are critical in controlling the effects of CP on the antioxidant activity of food products.

## 5. Conclusions

Cold plasma is a novel, non-thermal technology which has shown good potential for food decontamination. However, most of the research is largely focused on microbial inactivation studies, with limited emphasis on food quality. Cold plasma processing has been shown to affect the quality attributes of the food products during treatment as well as in storage. It presents a research opportunity to further explore the effects of cold plasma on the physico-chemical and sensory properties of the food products at the molecular level. The differences in the reported studies demonstrate the need for mechanistic studies to understand the interaction of plasma reactive species with food components. Optimization studies are also required to avoid the negative impacts on quality, such as accelerated lipid oxidation, loss of vitamins and sensory characteristics. The precise understanding of the mechanisms and control over the quality attributes will be required for cold plasma technology to realize its full potential at commercial scale.

## Figures and Tables

**Table 1:** Summary of effects of cold plasma processing on quality of food products.

Sample	Plasma	Quality Observation	Microbial Observation	References
Orange Juice	DBD, Air/MA65 (65% O_2_, 30% CO_2_, 5% N_2_), 90 kV, 30–120 s	No significant change in Brix or pH Vit.C is reduced by 22% in air PME activity reduced by 74% in air and 82% in MA65 Maximum total color difference is less than 1.2	Up to 5 log_10_ reduction of *Salmonella enterica*	[18]
Prebiotic orange juice	DBD, 70 kV (50 Hz), 15–60 s	Degradation of oligosaccharides in the juice Decrease in pH Increase in L* value and slight reduction in chroma and hue angle Decrease in total phenolic content and antioxidant capacity in some cases	NA	[19]
Cashew apple juice	PE-100, 80 kHz, N_2_, 10–50 mL/min, 5–15 min, 30 kPa	Decrease in vitamin C at higher flow rate Increase in sucrose content while glucose and fructose contents decreased Longer treatment promoted higher polyhphenol and total flavonoid content	NA	[20]
White grape juice	DBD, 60 Hz, 80 kV, 1–4 min, air	No significant change in pH, acidity and electrical conductivity of the juice An increase in non-enzymatic browning with minimal total color difference Decrease in total phenolics, total flavonoids, DPPH free radicals scavenging and antioxidant capacity An increase in total flavonols content	7.4 log_10_ CFU/mL reduction in *Saccharomyces cerevisiae* at 80 kV for 4 min	[21]
Pomegranate juice	Plasma jet, 25 kHz, Ar, 0.75–1.25 dm^3^/min, 3–7 min	Increase in total anthocyanin content No visual differences in color	NA	[22]
Radish sprouts	Microwave plasma, 2.45 GHz, 900 W, 669 Pa, 1–20 min, N_2_, 1 L/min	No change in color, water activity, ascorbic acid concentration and antioxidant activity Lower moisture content during storage	2.6 log_10_ reduction in *Salmonella typhimurium* 0.8 log_10_ reduction in total mesophilic aerobes	[23]
Blueberry	DBD, 50 Hz, 60–80 kV, 0–5 min, air	Decrease in firmness, total phenol, flavonoid and anthocyanin on extended cold plasma treatment at the higher voltage level Significant increase in total soluble solid No significant change in acidity and color (except fruit darkening at 80 kv for 5 min)	NA	[24]
Blueberry	Plasma jet, 47 kHz, 549 W, air, 4–7 cubic feet/min, 7.5 cm, 0–120 s	Significant reductions in firmness, color and anthocyanins at higher treatment times	Upto 2 log_10_ reduction in total aerobic plate count	[25]
Strawberry	DBD, 60 kV, 50 Hz, air, 5 min, indirect exposure	No significant change in color, firmness and respiration rate	2 log_10_ reduction in background microflora (aerobic mesophilic bacteria, yeast and mould)	[26]
Strawberry	DBD, 60 kV, 50 Hz, 65% O_2_ + 16% N_2_ + 19% CO_2_ and 90% N_2_ + 10% O_2_, 5 min, indirect exposure	Strawberries in high oxygen mixture showed higher firmness with similar respiration rates Some changes L* and a* values were observed	~3.0 log_10_ reduction in microbes in both gas mixtures	[27]
Mandarins	Microwave plasma, 2.45 GHz, 900 W, 1 L/min, 0.7 kPa, N_2_, He, N_2_ + O_2_ (4:1), 10 min	Increased total phenolic content and antioxidant activity No significant change in CO_2_ generation, weight loss, soluble solids, acidity, pH, ascorbic acid and color	Significant inhibition of *Penicillium italicum* (84% reduction in disease incidence)	[28]
Kiwifruit	DBD, 15 kV, 10–20 min	Improved color retention and reduced darkened area formation during storage No significant changes in color, hardness, vitamin C and antioxidant activity Longer treatment increase soluble solid content 15% decrease in chlorophyll a on day 0 with no difference on day 4	NA	[29]
Golden delicious apples	Gliding arc plasma, 60 Hz, air, 10–40 L/min, 1–3 min	No changes in color and texture	~3.5 log_10_ reduction in *Salmonella* and *E. coli* O157:H7 reduction	[30]
Apple (Pink Lady apples)	DBD, 12.7 kHz, 150 W, air, 30, 120 min	Up to 10% reduction of antioxidant content and antioxidant capacity No significant difference in total phenolic content but significant decrease in total phenolic index	NA	[31]
Melon	DBD, 15 kV, 12.5 kHz, air, 30, 60 min	No change in acidity, soluble solid content, dry matter, color and texture 17% and 7% reduction in peroxidase and PME activities respectively	3.4 and 2 log_10_ reductions in mesophilic and lactic acid bacteria respectively	[32]
Cherry tomatoes	DBD, 100 kV, 150 s, air	No significant difference in color, firmness, pH or total soluble solids	>5 and 3.5 log_10_ cfu/sample reduction in *E. coli* and *Listeria innocua* Up to 3.5 log_10_ cfu/sample reduction on spoilage microflora (mesophiles, yeast and mold)	[33]
Fresh fruit and vegetable slices (pears, cucumbers and carrots)	Plasma micro-jet, 30 mA, 500 V, 1–8 min	Less than 5% moisture loss in all three samples after 8 min treatment Minimal change in total color difference 3.6%, 3.2% and 2.8% reduction of vitamin C in cucumber, carrot and pear slice, respectively	90%, 60% and 40% *Salmonella* inactivation in carrot, cucumber and pear slice, respectively	[34]
Red chicory	DBD, 19.15 V, 3.15 A, 15 min, deionized water	No detrimental effects on color, freshness and texture Odor and overall acceptability slightly decreased during storage	>4 log_10_ cfu/cm^2^ reduction of *L. monocytogenes* and >5 log_10_ reduction of VTEC (*E. coli*)	[35]
Red chicory (radicchio)	DBD, 15 kV, 12.5 kHz, 15–30 min, air, 1.5 m/s	No significant effects on antioxidant activity and external appearance	1.35 log_10_ MPN/cm^2^ reduction of *E. coli* O158:H7 2.2 log_10_ cfu/cm^2^ reduction of *L. monocytogenes*	[36]
Romaine lettuce	DBD, 42.6 kV, 1.5 A, 10 min, air	No significantly change in the surface morphology, color, respiration rate and weight loss	0.4–0.8 log_10_ cfu/g reduction of *E. coli* O157:H7 in the leaf samples in the 1, 3, and 5 layer configurations 1.1 log_10_ cfu/g reduction in bulk stacking with 7 layers	[37]
Fresh produce (romaine lettuce, baby carrots and cocktail tomatoes)	Atmospheric pressure cold plasma, 3.95–12.83 kV, 60 Hz, Ar, 0.5–10 min	No significant changes in color in any samples	0.5, 1.7 and 1.5 log_10_ reduction of *E. coli* in carrot, tomato and lettuce, respectively	[6]
Lamb’s lettuce	Plasma jet, 7.12 MHz, 35 W, Ar, 20.000 sccm, 40 s	Strong reduction of phenolic acids and flavonoids Low levels of mono- and polyphenols in leaf after treatment Significant erosion of upper epidermis on leaf surfaces	NA	[38]
Unpeeled almond	Diffuse coplanar surface barrier discharge, 20 kV, 15 kHz, Air, O_2_, N_2_, CO_2_ and 90% CO_2_ + 10% Ar, 15 min	Plasma treatment with air and N_2_ resulted in a browning of the unpeeled almond surface color	>5.0, 4.8, 2.3, 3.0 and 2.0 log_10_ *Salmonella* Enteritidis PT30 reduction was observed for air, O_2_, CO_2_, CO_2_ + Ar and N_2_ plasma respectively	[39]
Black gram	Radio Frequency plasma, 2 Pa, air (0.15 mbar), 13.56 MHz, 30–50 W, 5–15 min	Surface etching and hydrophillization of surface Decrease in hardness, cooking time, ash and moisture content	NA	[40]
Brown rice	Radio Frequency plasma, air (0.15 mbar), 13.56 MHz, 40–50 W, 5–10 min	Decrease in cooking time, hardness, chewiness, contact angle, and moisture content Higher degree of gelatinization Increase in water uptake, L value and whiteness index	NA	[41]
Brown rice	DBD, 15 kHz, 250 W, air, 5–20 min	Decrease in pH and hardness Increase in L* and decrease in a* and b* values	Microbes studies: *Bacillus cereus*, *Bacillus subtilis*, *E. coli* O157:H7 and total aerobic bacteria 20 min plasma treatment resulted an approximately 2.30 log_10_ cfu/g bacterial reduction	[42]
Grains: wheat, bean, chick pea, soy bean, barley, oat, rye, lentil and corn	Low pressure cold plasma, 1 kHz, 20 kV, 500 mTorr, 300 W, air and SF_6_, 5–20 min	Slight change in moisture content of legume and wheat No difference in water soaking, yield and cooking time of legumes No change in wet gluten content, gluten index and sedimentation in wheat	3 log_10_ reduction of *Aspergillus* spp. and *Penicillum* spp. after 15 min treatment in SF_6_	[43]
Refined wheat flour	DBD plasma, 1–2.5 kV, 50 Hz, 1–5 min	No significant color change was observed on refined wheat flour	Significant increase in *Tribolium castaneum* (Herbst).	[44]
Wheat flour (soft and hard)	DBD, 60–70 kV, 5–10 min, air	An increase in the peak time, peak integral, elastic modulus, viscous modulus, dough strength and optimum mixing time No significant variation in tan σ for both flour	NA	[45]
Rice starch	Radio frequency plasma, 13.56 MHz, 40–60 W, 0.15 mbar, air, 5–10 min	Decrease in amylose content, turbidity, gelatinization temperature, retrogradation tendency, degree of starch hydrolysis and pasting temperature Increase in leaching of amylose, pasting, final viscosities, water absorption index, solubility, swelling power and syneresis	NA	[46]
Onion powder	Microwave plasma, 170 and 250 m Wm^−2^, 2.45 GHz, 400–900 W, 10–40 min, 0.7 kPa, He, 1 L/min,	No effect on color, antioxidant activity and quercetin concentration	2.1 log_10_ spores/cm^2^, 1.6 log_10_ spores/cm^2^ and 1.9 cfu/cm^2^ reduction of *Bacillus cereus*, *A. brasiliensis* spore, and *E. coli* O157:H7, respectively	[47]
Bacon	Atmospheric pressure plasma, 75–125 W, 13.56 MHz, 60 s and 90 s, He (10 lpm) and He + O_2_ (10 lpm and 10 sccm)	Increase in L* value No change in pH Lower TBARS values at day 0, while after 7 days of storage, plasma treated samples had higher TBARS value than control	Pathogens studied: *Listeria monocytogenes*; *Escherichia coli* and *Salmonella typhimurium* Helium plasma reduce the pathogens in 1–2 log_10_ range Helium/oxygen gas mixture shows a reduction of pathogen in a range of 2–3 log_10_ 4.53 log_10_ cfu/g reduction in total aerobic count	[48]
Fresh and frozen pork	Plasma jet, Air, 20 kV, 58 kHz, 1.5 amp,0–120 s	No significant changes in volatile basic nitrogen, peroxide value and TBARS No significant impact on the sensory characteristics on frozen pork Significant changes in color for both fresh and frozen pork	1.5 log_10_ reduction of *E. coli* O157:H7 >1.0 log_10_ unit *Listeria monocytogenes*	[49]
Fresh pork	Microwave plasma, air, 5–10 min, 2.45 GHz, 1.2 kW, 20 slm	Increased a value and decreased b values of pork meat Difference in reflectance and fluorescence. Significant changes in pH	Aerobic viable count remained between 10^2^ and 10^3^ cfu/g during the storage period of 20 days	[50]
Fresh pork and beef	Thin-layer DBD plasma, 1–10 min, 100 W, N_2 _+ O_2_	No significant effect on texture, L* and b* value Decrease in a* values after 5 min exposure Significant lipid oxidation after 10 min exposure No change sensory parameters except taste, which was negatively influenced	Up to 2.7 log_10_ cfu/g reduction of *Listeria monocytogenes*, *E. coli* O157:H7 and *Salmonella tryphimurium* in pork and beef	[51]
Pork Loin	DBD, He or He + 0.3% O_2_, 5–10 min, 3 kV, 30 kHz, 10 slm	Decrease in pH and L* values with no change in a* and b* values Higher lipid oxidation in Helium- oxygen plasma Significant reductions in sensory quality parameters (appearance, color, odor, acceptability)	Up to 0.55 log_10_ reduction of *E. coli* reduction in helium and helium-oxygen plasma Up to 0.59 log_10_ reduction of *L. monocytogenes* reduction in helium and helium-oxygen plasma	[52]
Beef jerky	RF plasma, Ar, 20,000 sccm, 200 W, 0–10 min	No significant change in fatty acid composition, color and shear force	1.8 log_10_ reduction in *Staphylococcus aureus* after 8 min treatment	[8]
Pork	Pulsed plasma, 0.8 MPa, 20–100 kHz, 1.2 kVA, N_2_, He, Ar	No significant differences in color and pH	Up to 3 log_10_ cfu/cm^2^ reduction of psychrotroph bacteria, yeast and mold	[53]
Raw pork	Low-pressure plasma, 0–10 min, He, 20 kPa	Significant changes in total color difference, hue angle and chroma Decreased Ferric reducing ability after 14 days of storage 3% increase in polyunsaturated fatty acids during storage No oxidative processes were observed	NA	[54]
Ground pork	Plasma jet, 7 kV, 25 kHz, 600 W, 1.67 × 10^−4^ m^3^/s, 60 min	Increase in nitrite content from 0.64 to 60.50 mg/kg No difference from control in nitrosyl hemochrome, color, residual nitrite, texture, lipid oxidation and protein oxidation Higher score in taste and overall acceptability	No effect on total aerobic count	[55]
Fresh mackerel fillets	DBD, 70–80 kV, 50 Hz, air, 1–5 min	No changes in pH, color (except decrease in L* value), fat and moisture content Higher oleic and eicosapentaenoic acid in plasma treated samples Significant primary oxidation (PV and Dienes) No significant difference in TBARS values. Decrease in T_21_ (dense myofibrillar network) with increased T_22_ (extramyofibrillar water)	No significant reduction in the total aerobic mesophilic count Significant reduction in psychotropic bacteria, lactic acid bacteria and Pseudomonas	[56]

Vit.C: vitamin C; MA: Modified atmosphere; NA: Not available; DBD: Dielectric barrier discharge; PME: Pectin methylesterase; TBARS: Thiobarbituric acid reactive substance; PV: peroxide value.

**Table 2:** Effects of cold plasma on enzymes in food. Adapted from [2], with permission.

Enzyme	Food Product	Plasma	Salient Results	Reference
Polyphenol oxidase	Fresh-cut apples	DBD, 15 kV, 12.7 kHz, 10–30 min, Air, 1.5 m/s	Linear decrease in activity with treatment time. Residual activity of 88%, 68% and 42% after 10, 20 and 30 min of treatment.	[66]
Polyphenol oxidase	Fresh-cut apples	DBD, 150 W, 15 + 15, 30 + 30 min, Air, 1.5 m/s	Noticeable reduction in superficial browning but not proportional to treatment time Variable effects on PPO activity Effect were strictly cultivar dependent	[67]
Peroxidase	Fresh-cut melon	DBD, 15 kV, 12.5 kHz, 15 + 15, 30 + 30 min, Air	Residual activity were 91% and 82% after 15 + 15 and 30 + 30 min treatment, respectively	[32]
Pectin methylesterase	Fresh-cut melon	DBD, 15 kV, 12.5 kHz, 15 + 15, 30 + 30 min, Air	15 + 15 min treatment was ineffective Residual activity was 94% after 30 + 30 min treatment.	[32]
Superoxide dismutase	Mushrooms (*Agaricus bisporus*)	Plasma jet, 18 kV, 10 kHz, 98% Ar + 2% O_2_, 5 L/min	SOD activity was higher in plasma treated mushroom during storage	[68]

SOD: Superoxide dismutase; PPO: Polyphenol oxidase.
